# Comprehensive analysis of gene regulation network and immune signatures of prognostic biomarker YAP1 in pancreatic cancer

**DOI:** 10.7150/jca.49117

**Published:** 2020-10-08

**Authors:** Yuan Yang, Ya Zheng, Xin Liu, Rui Ji, Zhaofeng Chen, Qinghong Guo, Guozhi Wu, Yuping Wang, Yongning Zhou

**Affiliations:** 1 The First Clinical Medical School, Lanzhou University, Lanzhou, 730000, PR China.; 2 Department of Gastroenterology, The First Hospital of Lanzhou University, Lanzhou 730000, PR China.; 3 Key Laboratory for Gastrointestinal Diseases of Gansu Province, The First Hospital of Lanzhou University, Lanzhou 730000, PR China.; 4 Department of Gastroenterology, The 940th Hospital of Joint Logistic Support Force of PLA, Lanzhou 730050, China.

**Keywords:** pancreatic cancer, YAP1, immune infiltration

## Abstract

**Background:** Pancreatic cancer (PC) is one of the most common digestive malignancy, with severe cancer-related death and disease burden. Yes-associated protein 1 (YAP1) has been reported to be involved in the tumorigenesis and progression of several cancers, thus leading to poor prognosis of patients. However, the relationship between YAP1 and immune microenvironment in PC deserve more scrutiny.

**Methods:** GEPIA, OncoLnc, PROGgeneV2 and HPA database were utilized to analyze the expression (transcriptome and protein levels) and overall survival of YAP1 in PC. Then, we evaluated the risk factors associated with overall survival based on public data from TCGA-PAAD via Cox regression. Besides, LinkedOmics was utilized to identify co-expression genes and the potential regulation network of YAP1. Furthermore, we explored the relationship between YAP1 and immune infiltration using CIBERSORT algorithm and GEPIA database.

**Results:** The age, lymph node metastasis status and up-regulated YAP1 expression have been proved to be independent prognostic factors for poor prognosis. The functions of YAP1 and co-expression genes were mainly involved in the angiogenesis, immune response-regulating signaling pathway, regulation of actin cytoskeleton, NOD-like receptor signaling pathway and cytokine-cytokine receptor interaction. Specifically, increased YAP1 expression was significantly correlated with immune infiltrating levels of resting CD4^+^T cells.

**Conclusions:** Our findings provide evidence of the immune regulatory role of YAP1 in PC and help elucidate the role of YAP1 in carcinogenesis as well.

## Introduction

Pancreatic cancer (PC) is one of the most common digestive malignancy, with severe cancer-related death and disease burden 1,2. Although various new diagnoses and treatments have been achieved for the management of PC, the prognosis remains unsatisfactory due to the late detection, chemotherapeutic resistance and postoperative recurrence 3-5. Recently, some therapeutic targets in PC, especially kinase and immune checkpoint inhibitors, have been found with clinical significance 6. However, these therapeutic targets played a limited role in PC management, and only BRCA-mutant pancreatic cancers have confirmed favorable response to Olaparib 7. Therefore, identification of more promising therapeutic targets for PC could facilitate individualized treatment.

Yes-associated protein 1 (YAP1), the transcriptional effector of the Hippo signaling pathway, acts as a potential oncogene in various types of malignant tumors 8-12. Previous studies have indicated dynamics of YAP1 expression could promote malignant transformation, enhance the expansion of several cancer stem-like cells and chemotherapy drug resistance 13. Through pharmacologic or genetics inhibition of YAP1 could not only suppress malignant transformation of relevant cancers but also provide an improved measure of drug sensitivity to chemotherapy 13. These results suggested that targeting YAP1 might be a novel therapeutic strategy. However, the unique immunosuppressive microenvironment and poor T cell infiltration have become underlying challenges in the treatment of PC, thus leading to its lethality 14,15. Although several immunotherapies such as immune checkpoint blockade or engineered T cells could be the promising strategies to PC, there was no substantial improvement in the treatment of PC 16,17. Thus, the relationship between YAP1 and immune microenvironment in PC deserve more scrutiny.

In this study, we performed a systematic analysis of the potential value of YAP1 in PC. GEPIA, OncoLnc, PROGgeneV2 and HPA database were utilized to analyze the expression and overall survival of YAP1. Meanwhile, the correlation of YAP1 with the prognosis of PC was further evaluated based on public data from TCGA via Cox regression analysis. To better understand YAP1 co-expression genes and potential regulation network that could underlie PC development, we performed LinkedOmics analysis along with Gene Ontology (GO) and Kyoto Encyclopedia of Genes and Genomes (KEGG). Furthermore, we explored the relationship between YAP1 and tumor immune infiltration using CIBERSORT algorithm and GEPIA database. Our results could provide a fresh perspective on the mechanisms underlying PC.

## Materials and Methods

### Data download and preprocessing

The mRNA expression profiles (HTSeq—FPKM) and corresponding clinical data of 178 patients were extracted from TCGA data portal with the closing date of 25 April 2020. Subsequently, we conducted data preprocess to obtain complete clinical information for further investigation. Finally, 172 cases with eligible clinical information were performed to univariate and multivariate regression analysis.

### YAP1 expression and overall survival analysis of PC by GEPIA, PROGgeneV2 and HPA

GEPIA (http://gepia.cancerpku.cn/) and OncoLnc (http://www.oncolnc.org) are public online databases for visualization and analysis of the standard genomic datasets from the TCGA and/or the GTEx projects 18,19. In this study, the correlation between such as gene expression analysis, clinicopathological factors analysis and overall survival analysis of YAP1 was assessed in patients with PC. GSE57495 was performed to validate the outcomes via the PROGgeneV2 platform 20. The HPA database (www.proteinatlas.org) was utilized to analyze the protein expression of YAP1 between healthy control and pancreatic cancer as measurements of RNA levels 21.

### Interrelated pathways analysis of YAP1 and its co-expression genes by LinkedOmics and GeneMINIA

LinkedOmics (www.linkedomics.org) is a unique online analytical platform to provide comprehensive multi-omics data analysis 22. The TCGA-PAAD datasets and corresponding clinical data were obtained firstly. Subsequently, the Link Finder module based was utilized to analyze the co-expression genes of YAP1 in PC with FDR of 0.05. GSEA was performed to enrichment analysis of YAP1 co-expression genes in PC, with a minimum number of genes (size) of 3, the simulation of 1000 and an FDR of 0.05. Finally, GeneMINIA was utilized to construct the PPI network and detect the fundamental functions of these genes 23.

### Immune landscape related to YAP1 expression level

CIBERSORT was utilized to evaluate the relevance of gene expression and 22 tumor-infiltrating immune cells (TIICs) in cancer 24. The tumor samples were divided into a low expression group (YAP1^low^) and a high expression group (YAP1^high^) based on the median expression of YAP1. The screening criteria were determined as 1000 permutation and *P*-value < 0.05, respectively. Afterward, the fractions of immune cells produced by CIBERSORT were subsequently analyzed. 'Correlation' immune-related module of GEPIA was used to validate the outcomes.

### Statistical analysis

The analyses were conducted using the 'R' software (version 3.6.3). The univariate and multivariate Cox regression analysis was performed to identify overall survival-related risk factors in the TCGA-PAAD projects. *P-*value < 0.05 was considered to have significant statistical significance.

## Results

### High expression of YAP1 correlated with unfavorable prognosis in patients with PC

**Figure 1:**
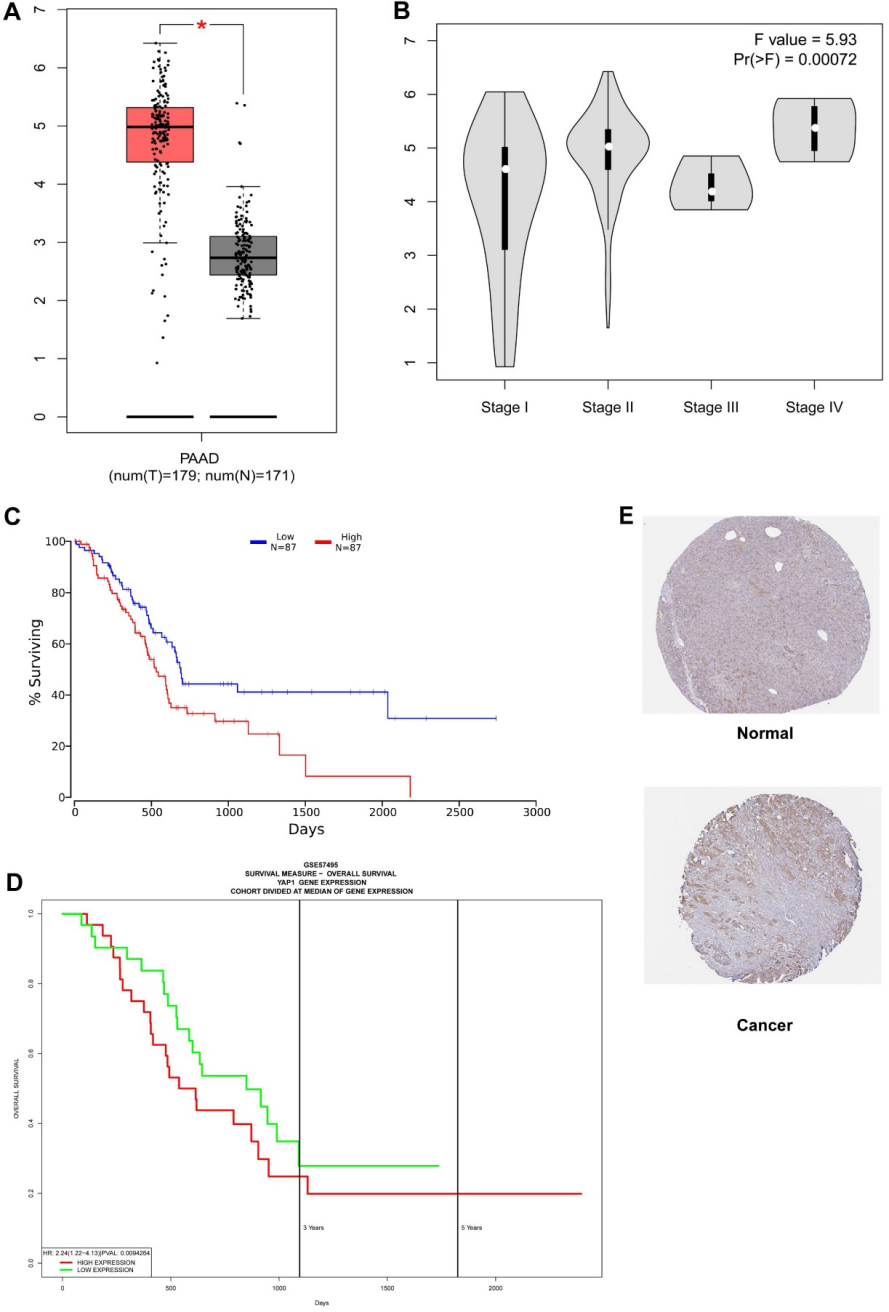
Survival outcomes and expression differences analyzed by GEPIA, OncoLnc, PROGgeneV2 and HPA. (A) Differential expression of YAP1 in PC tissue and normal tissue. (B) Significant differences in YAP1 expression in different pathological stages. (C) The increase of YAP1 expression was correlated with a poor prognosis. (D) Survival analysis results from PROGgeneV2 for verification. (E) The HPA database was utilized to analyze the protein expression of YAP1 between healthy control and PC as measurements of RNA levels.

**Figure 2:**
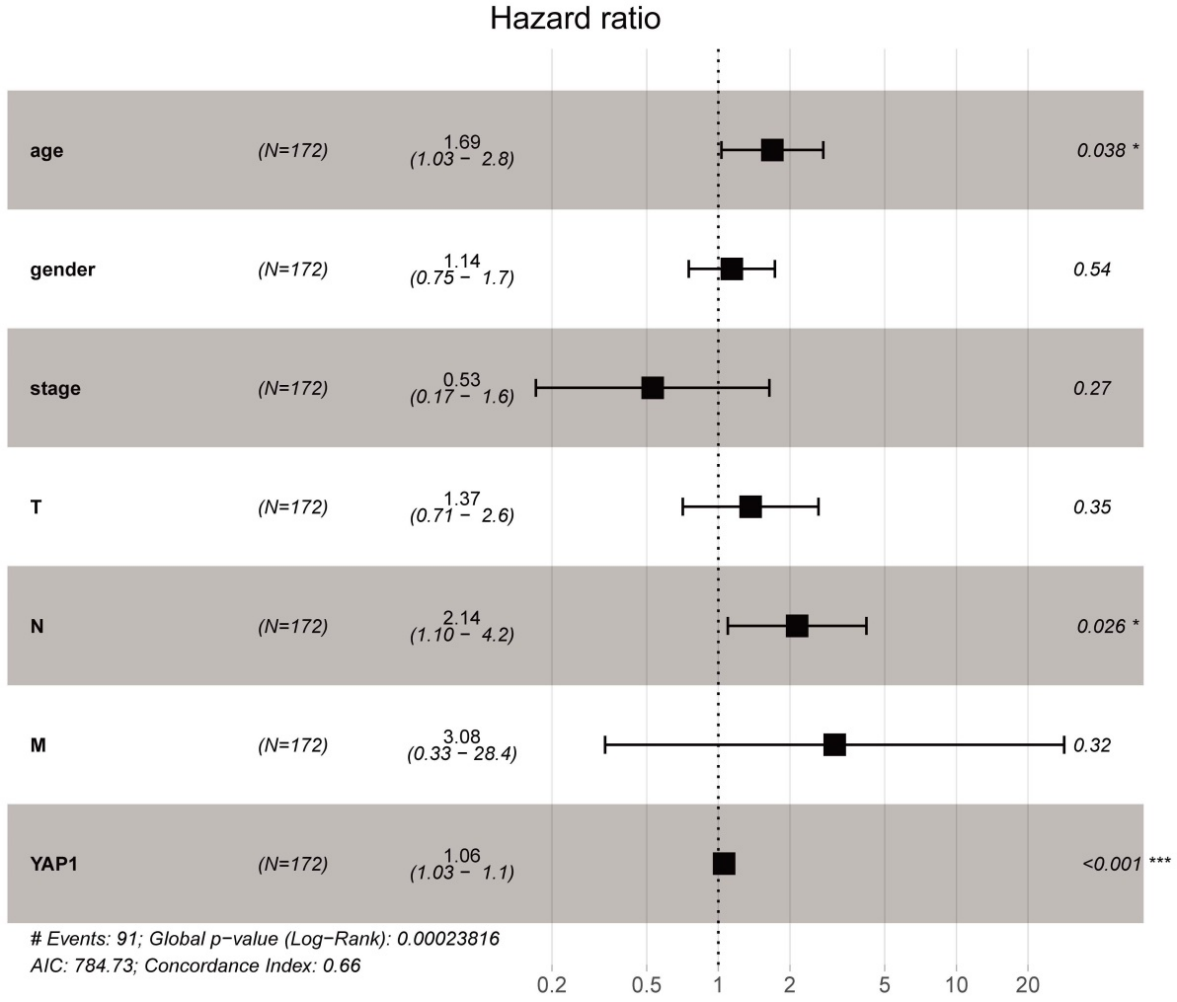
Multivariate Cox analysis of YAP1 expression and other clinicopathological variables. The age, lymph node metastasis status and up-regulated YAP1 expression are independent prognostic factors of poor prognosis.

As shown in Fig. 1A, YAP1 expression was notably higher in the PC compared to normal tissues (*P*-value < 0.05). In addition, increased expression of YAP1 was markedly correlated with the advanced pathological stage (Fig. 1B, *P*-value < 0.001) and poor overall survival (Fig. 1C). The same overall survival analysis result of GSE57495 was verified on the PROGgeneV2 platform (Fig. 1D). Moreover, univariate Cox regression analysis indicated that the T stage, lymph node status and the expression of YAP1 are notably correlated with overall survival (Table 1). In multivariate Cox analysis (Table 1, Fig. 2), the age, lymph node metastasis status and up-regulated YAP1 expression are independent prognostic factors of poor prognosis.

### Co-expression genes of YAP1 and relevant enrichment analysis in patients with PC

**Figure 3:**
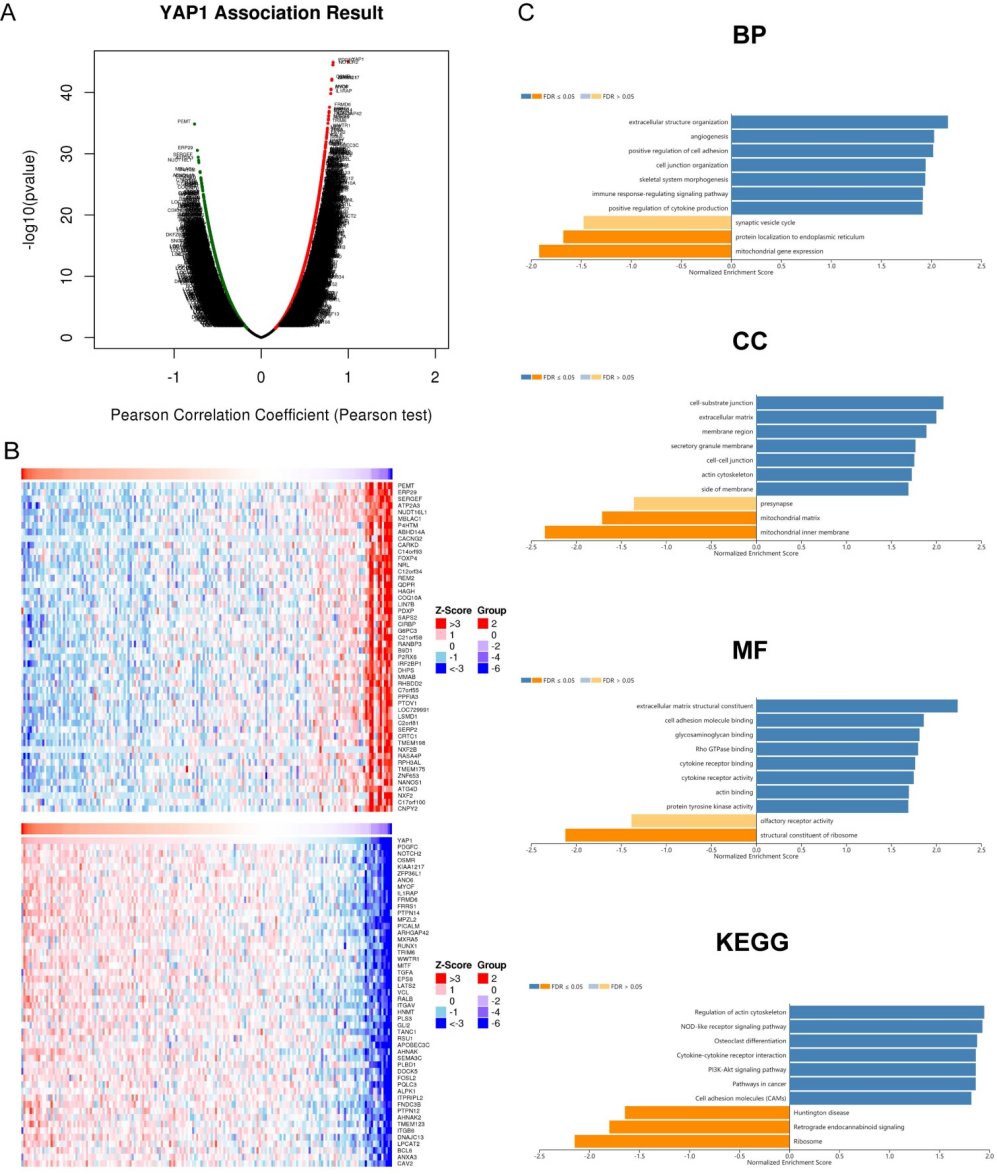
YAP1 co-expression genes in PC (LinkedOmics). (A) The genes identified by Pearson test positively and negatively correlated with YAP1 in PC. (B) Heat maps showing top 50 genes positively and negatively correlated with YAP1 in PC. Red dots were positively correlated with YAP1, while green dots were negatively associated with YAP1. (C) Enrichment Go and KEGG analysis of YAP1 in PC.

To further clarify the significance of YAP1 in PC, we analyzed co-expression gene sets of YAP1 and further explored their potential roles using the data of TCGA-PAAD projects with LinkedOmics. As shown in Fig. 3A, 6,651 genes (dark red dots) were positively correlated with YAP1, while 5,151 genes (dark green dots) were negatively correlated with YAP1 in PC (FDR<0.05). Besides, the top 50 genes significantly correlated with YAP1 were shown in Fig. 3B. YAP1 expression showed a significant positive association with expression of PDGFC (cor = 0.826, FDR = 1.22E-41), NOTCH2 (cor = 0.824, FDR = 2.10E-41), OSMR (cor = 0.811, FDR = 3.21E-39) and KIAA1217 (cor = 0.810, FDR = 3.21E-39).

Functional Enrichment Analysis was subsequently conducted. GO term showed that YAP1 and its co-expression genes were mainly involved in the immune response-regulating signaling pathway, positive regulation of cytokine production, positive regulation of cell adhesion, extracellular structure organization and angiogenesis. KEGG pathway analysis of these genes showed enrichment in the Regulation of actin cytoskeleton, NOD-like receptor signaling pathway, Cytokine-cytokine receptor interaction, PI3K-Akt signaling pathway and Pathways in cancer.

### YAP1 networks of kinase, miRNA or transcription factor targets in PC

**Figure 4:**
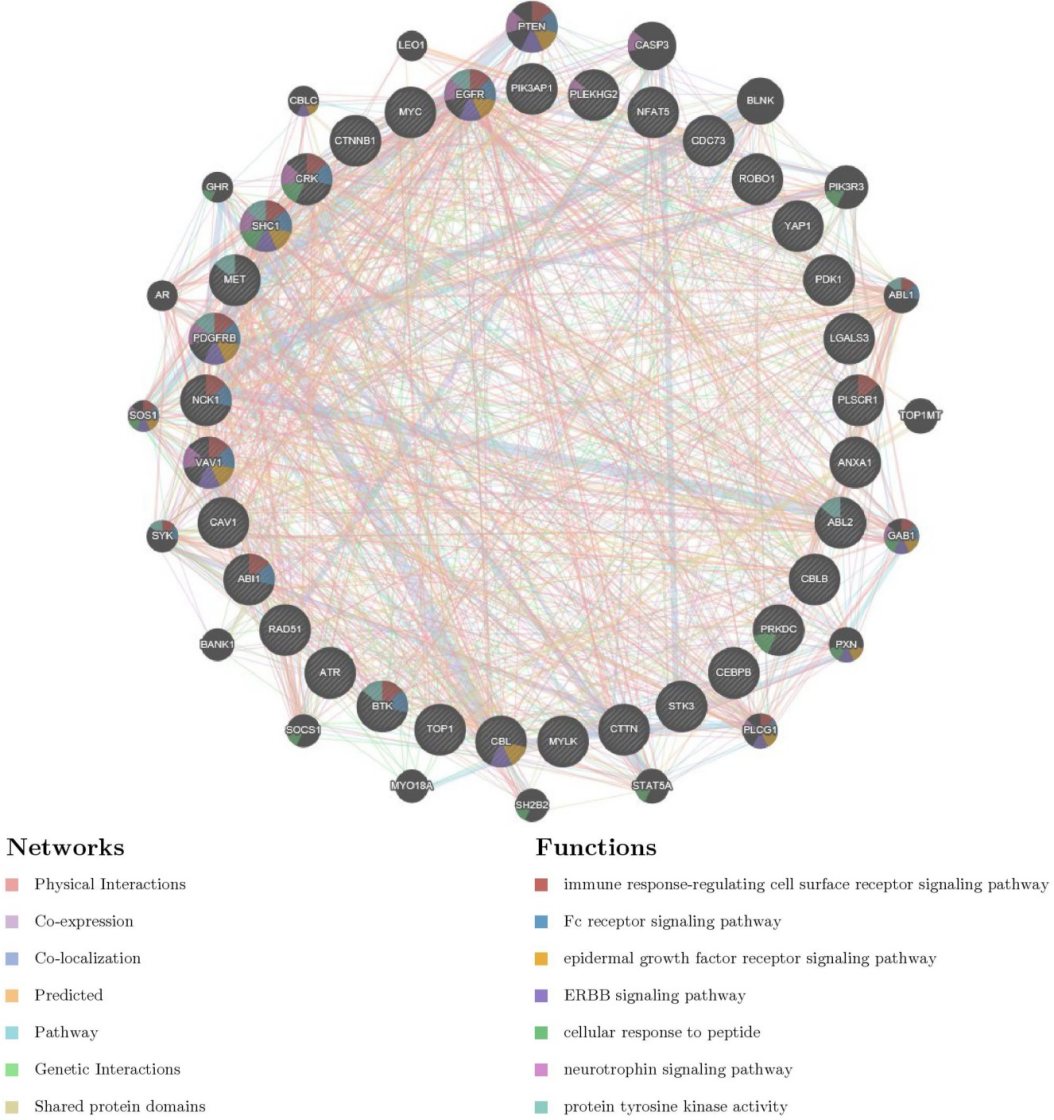
PPI network of ABL1 kinase-target networks (GeneMANIA). The biological functions of the gene sets of ABL kinase-target networks was assessed using the applied bioinformatics methods via PPI network.

To further explore the potential regulators of YAP1 in PC, we explored networks of kinase, miRNA or transcription factor (TF) targets enrichment of YAP1 co-expression genes. As a consequence, kinases ABL1, LYN, MAPK1, PRKCA and CDK1 were shown as the top 5 most significant targets (Table 2). PPI network was further constructed, and the results indicated that regulation of these genes were involved in the immune-related pathway (Fig. 4). Moreover, the miRNA network targets (MIR-26A, MIR-26B, MIR-186, MIR-374, MIR-200B, MIR-200C, MIR-381) and the TF network targets (V$IRF_Q6, V$SRF_Q6, V$PEA3_Q6, V$CEBP_Q2_01, V$AREB6_04) were shown in Table.2 and Supplementary material. As expected, the PPI network of both indicated the involvement of the immune-related pathway.

### Immune Cell Infiltration of YAP1 in patients with PC

**Figure 5:**
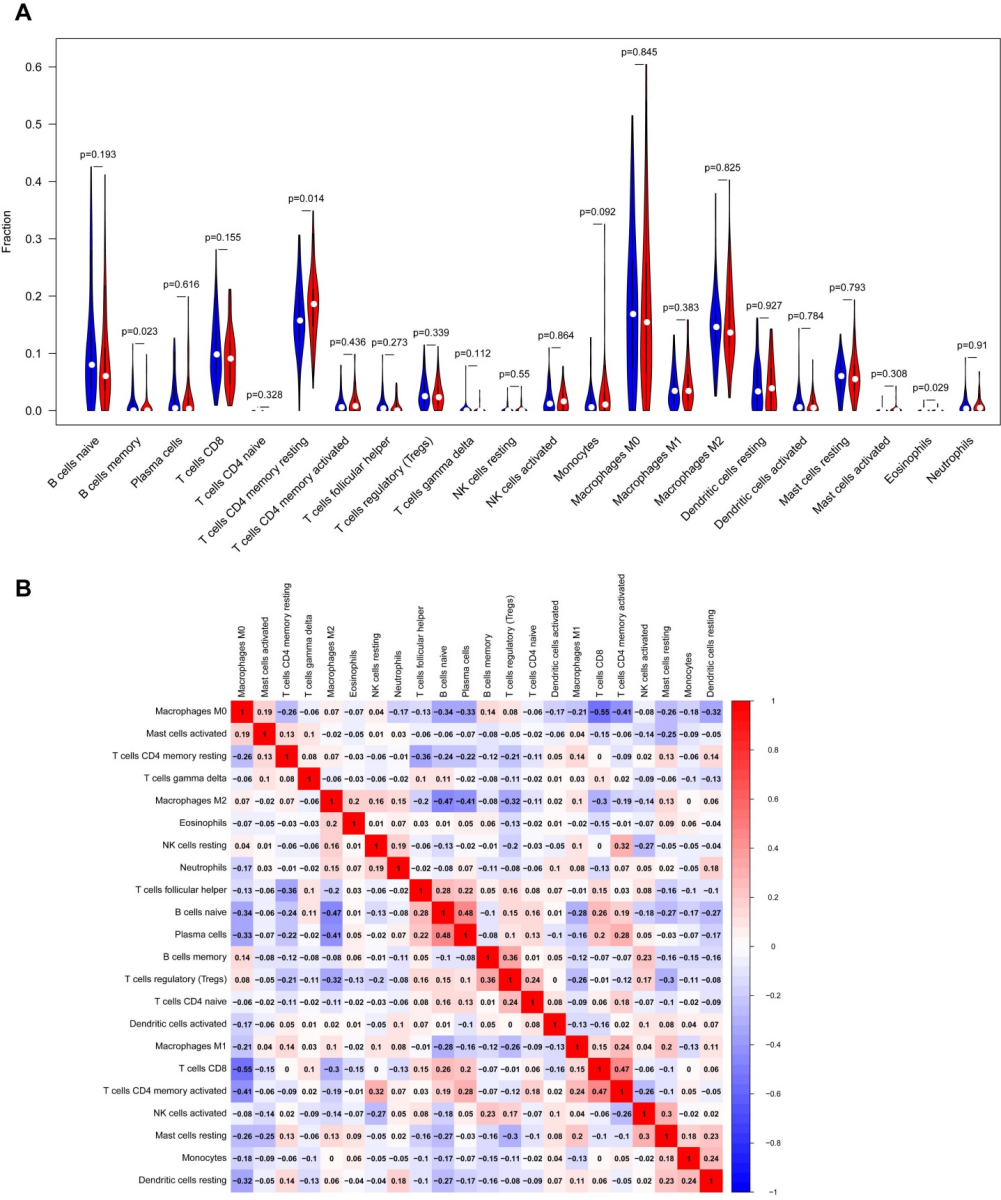
Analysis of immune infiltration level with YAP1 high and low expression groups in PC. (A) Violin plot of immune infiltration level between YAP1 high (red plot) and low expression groups (blue plot). (B) The proportions of different TIICs subpopulations were weakly to moderately correlated.

Our study demonstrated the proportions of immune cells changed significantly among different samples. The infiltration of M0, M2 macrophages and resting memory CD4^+^T cells showed a relatively higher abundance compared to other immune cells (as shown in Fig. 5A) Besides, our study indicated that the fractions of resting memory CD4^+^T cells, B cells memory and Eosinophils in YAP1^high^ group were notably higher than that in YAP1^low^ group PC patients, implying the possible roles of these immune cells in YAP1-dependent manner (Fig. 5A) As shown in Fig.5B, the correlation heatmap revealed a weak to moderate correlation within the proportions of different TIICs subpopulations. 'Correlation' module of GEPIA was utilized to assess the correlation between YAP1 expression and cell surface markers of different types of TIICs (Table 3). Spearman correlation coefficient was conducted and the results indicated that YAP1 might play a crucial role in regulating the abundance of Th2, Tfh, Th17, T cell exhaustion and Mast cell. Whether YAP1 is an important factor for B cell and Eosinophils immune infiltration needs more experimental evidence.

## Discussion

Accumulating evidence has demonstrated that several cancer genes and its related signaling pathways, such as Hippo/YAP signaling, are involved in the tumorigenesis and progression of PC 25. As essential components of Hippo/YAP signaling, YAP1 has been reported to exert a notable drug resistance role in various cancers as well 13. The unique characteristics of the tumor microenvironment in PC have been regarded as one of the significant reasons that led to conventional chemotherapy drugs and immunotherapy resistance 25. However, the correlation of YAP1 and the tumor microenvironment in PC is not completely clarified yet. Therefore, we performed a comprehensive bioinformatics analysis to explore the potential roles of YAP1 and its immunoregulatory network in PC.

We first analyzed the correlation between the transcriptome level of YAP1, pathological stage along with prognosis. The results indicated that YAP1 was up-regulated in PC significantly. Besides, high expression of YAP1 was significantly correlated with pathological stage and poor survival of PC patients. Multivariate regression analysis showed that age, lymph node metastasis status and the mRNA level of YAP1 were independent prognostic factors. Previous research evaluated the association between YAP1 and lymph node metastasis status, and relevant study indicated that YAP-dependent metabolic adaptation contributed to tumor metastasis to lymph nodes 26.

Co-expression network analysis was widely used for characterization of important modules and interpretation of its biological function. We next utilized GO term and KEGG pathway analysis to explore the functions of the top 50 genes significantly correlated with YAP1 in PC. Our data revealed YAP1 and associated co-expression genes were primarily involved in angiogenesis, immune response-regulating signaling pathway, regulation of actin cytoskeleton, NOD-like receptor signaling pathway and cytokine-cytokine receptor interaction. These findings suggested that YAP1 played a crucial role in tumorigenesis and progression. The previous study indicated YAP/TAZ as effectors of VEGF signaling was involved in aberrant angiogenesis, thereby prompting progression and cancer metastasis 27. Besides, YAP1 contributed to cancer distant metastasis via regulation of actin dynamics as well 28. The NOD-like receptor signaling pathway has been demonstrated to increase the incidence of cancer emergence, and its cascade was involved in neovascularization, metastasis, and other immunosuppressive functions 29. We can infer that oncogene YAP1 is crucial in regulating pancreatic cells through the NOD-like receptor signaling pathway.

Furthermore, cytokine-cytokine receptor interaction is linked with immunosuppression 30. Recent research revealed that how the secretion of inflammatory cytokines and its cascade could promote tumor-induced immune suppression, thereby resulting in rapid tumor progression 31. This process suggested a possible connection with the interference of cytokine-cytokine receptor interaction caused by altered YAP1. With a better understanding of the biological functions of YAP1 would help verify the detailed processes and pathways.

For further mining important driving factors for cancers, we explored the network of Kinases, miRNA and TF potentially responsible for YAP1 dysregulation. We found that Kinase_ABL1, LYN, MAPK1, PRKCA and CDK1 were the top5 Kinases associated with YAP1. Interestingly, our analysis of the network of Kinases suggested that primary Kinases contributed to the immune response-regulating cell surface receptor signaling pathway as well. Therefore, the potential role of immune response deserved further experimental verification. Subsequently, the network of miRNA and TF were analyzed in the same ways, respectively. Both of them were related to the immune-related pathway. These results powerfully revealed the necessity of exploring the immune functions of YAP1.

Accumulating evidence indicated the immune cell infiltration had a strong influence on the progression and metastasis of cancers, thereby affecting the prognosis of relevant cancer 32-35. Besides, immune-related pathways in diseases are considered as a potential target for cancer therapy 36. A recent study suggested myeloid-derived suppressor cells (MDSC) was identified as the major infiltrating immune cell type in prostate cancer 37. Activation of the Hippo-YAP signaling pathway driven by the YAP-TEAD complex could promote MDSC recruitment, thereby involving in cancer progression 37. The results from CIBERSORT indicated that YAP1 expression was correlated with immune infiltration levels of resting memory CD4^+^T cells, B cells memory and Eosinophils. The association of YAP1 with resting CD4^+^T cells was validated in GEPIA database. Our findings revealed that resting CD4^+^T cells were found at increased levels in the high YAP1 expression group, and we could infer a possible mechanism where YAP1 regulated the functions of resting CD4^+^T cells in PC. Whether YAP1 is an important factor for B cell memory and Eosinophils immune infiltration needs more experimental evidence. Interestingly, the regulatory TF network of YAP1 and its co-expression genes was involved in response to type I interferon. The previous study has demonstrated the immunomodulatory role of type I IFNs in shaping T cell responses 38. Therefore, together these findings indicate that YAP1 plays a crucial role in the regulation and recruitment of immune infiltrating cells in PC.

Although YAP1 may act as an important immune checkpoint in PC, there are several limitations in our study. First, the samples analyzed in our study are obtained from TCGA, and the role of racial diversity has not yet been elucidated. Second, experimental study should be conducted in another independent cohort to validate our results. Furthermore, the relationship between YAP1 and molecular subtypes of pancreatic cancer is unknown, which may affect our results.

In summary, we demonstrated that the interaction between YAP1 and immune function might be mediated through immune infiltration of resting CD4^+^T cells. Our results suggested that activation YAP1 dependent immune regulatory network may enhance the activity of resting CD4^+^T cells, thereby promoting tumor progression in PC. Together these findings indicate that YAP1 may be a potential therapeutic target, which provides a refreshing perspective on the mechanisms underlying PC and disease management.

## Supplementary Material

Supplementary figures and tables.

## Figures and Tables

**Table 1:** Univariate and Multivariate Cox regression analyses of clinical characteristics and survival in patients with PC

Clinical variable	Univariate analysis		Multivariate analysis	
	HR (95%CI)	*P*-value	HR (95%CI)	*P*-value
Age	1.353 (0.853-2.145)	0.199	1.686 (1.030-2.758)	0.038
Gender	1.214 (0.804-1.834)	0.356	1.139 (0.751-1.728)	0.540
Stage	1.318 (0.901-1.928)	0.155	0.530 (0.171-1.639)	0.270
T	1.569 (1.013-2.432)	0.044	1.367 (0.709-2.635)	0.350
N	2.324 (1.380-3.912)	0.002	2.142 (1.096-4.187)	0.026
M	0.905 (0.285 -2.871)	0.866	3.08 (0.335-28.397)	0.320
YAP1	1.052 (1.025-1.080)	<0.001	1.057 (1.025-1.089)	<0.001

**Table 2:** The Kinase, miRNA and transcription factor-target networks of YAP1 in PAAD (LinkedOmics)

Enriched Category	Gene set	LeadingEdge Num	FDR
Kinase target	Kinase_ABL1	33	0.005
	Kinase_LYN	26	0.007
	Kinase_MAPK1	91	0.006
	Kinase_PRKCA	88	0.008
	Kinase_CDK1	92	0.01
miRNA target	TACTTGA,MIR-26A, B	126	5.54e-04
	ATTCTTT,MIR-186	92	0.001
	TATTATA,MIR-374	121	0.002
	CAGTATT,MIR-200B, C	167	0.003
	CTTGTAT,MIR-381	80	0.003
Transcription Factor	V$IRF_Q6	116	4.96e-04
	V$SRF_Q6	100	0.002
	V$PEA3_Q6	124	0.003
	V$CEBP_Q2_01	97	0.002
	V$AREB6_04	89	0.003

**Table 3:** A correlation analysis was performed between the gene markers expressed in immune cells and the expression of YAP1 using the 'Correlation' module of GEPIA

Description	Gene markers	Pancreas			
		Tumor		Normal	
		R	P	R	P
CD8^+^T cell	CD8A	0.25	0.00084	0.58	1.4e-16
	CD8B	0.19	0.0094	0.54	1.3e-14
T cell (general)	CD2	0.21	0.0042	0.62	9.3e-20
	CD3E	0.2	0.008	0.62	3.2e-19
B cell	CD19	0.11	0.14	0.34	4.6e-06
	CD79A	0.12	0.11	0.29	0.00015
Natural Killer cell	KIR2DL1	0.14	0.07	0.069	0.37
	KIR2DL3	0.2	0.0077	0.25	0.00087
	KIR2DL4	0.23	0.0016	0.27	0.00045
	KIR3DL1	-0.028	0.71	0.27	0.00033
	KIR3DL2	0.17	0.019	0.12	0.13
	KIR3DL3	0.13	0.074	0.044	0.57
	KIR2DS4	0.087	0.25	0.24	0.0016
Neutrophils	CD66b	0.16	0.031	0.11	0.15
	CD11b	0.39	9.30e-08	0.61	4.2e-19
	CCR7	0.11	0.16	0.53	6.3e-14
Th1	T-bet	0.13	0.085	0.49	1.3e-11
	STAT4	0.062	0.41	0.7	3.2e-26
	TNF-α	0.11	0.13	0.45	9.5e-10
Th2	GATA3	0.28	0.00014	0.38	2.6e-07
	STAT6	0.42	7.4e-09	0.79	3e-37
	STAT5A	0.38	1.4e-07	0.69	1.4e-25
	IL13	-0.12	0.11	0.18	0.019
Tfh	BCL6	0.64	3.1e-22	0.73	3.2e-30
Th17	STAT3	0.64	2.8e-22	0.76	1.5e-33
	IL17A	0.11	0.16	0.085	0.27
T cell exhaustion	PD-1	0.18	0.016	0.43	5.3e-09
	CTLA4	0.23	0.016	0.44	1.4e-09
	LAG3	0.049	0.52	0.53	4.8e-14
	TIM-3	0.4	3.1e-08	0.7	2.3e-26
Mast cells	TPSB2	0.24	0.0014	0.29	0.00015
	TPSAB1	0.29	7e-05	0.33	1.3e-05
	CPA3	0.43	1.7e-09	0.24	0.0018
	MS4A2	0.4	2.6e-08	0.31	3.6e-05
	HDC	0.26	0.00054	0.39	1.8e-07

## References

[1] Bray F, Ferlay J, Soerjomataram I (2018). Global cancer statistics 2018: GLOBOCAN estimates of incidence and mortality worldwide for 36 cancers in 185 countries. CA Cancer J. Clin.

[2] Chen X, Yi B, Liu Z (2020). Global, regional and national burden of pancreatic cancer, 1990 to 2017: Results from the Global Burden of Disease Study 2017. Pancreatology.

[3] Daamen LA, Groot VP, Intven MPW (2019). Postoperative surveillance of pancreatic cancer patients. Eur J Surg Oncol.

[4] Conroy T, Ducreux M (2019). Adjuvant treatment of pancreatic cancer. Curr Opin Oncol.

[5] Chen L, Shi S, Meng Q (2017). Complex roles of the stroma in the intrinsic resistance to gemcitabine in pancreatic cancer: where we are and where we are going. Exp Mol Med.

[6] Osipov A, Saung MT, Zheng L (2019). Small molecule immunomodulation: the tumor microenvironment and overcoming immune escape. J Immunother Cancer.

[7] Golan T, Hammel P, Reni M (2019). BRCA Maintenance Olaparib for Germline-Mutated Metastatic Pancreatic Cancer. N Engl J Med.

[8] Jaffer AA, Xu Y, Huo L YAP1 mediates gastric adenocarcinoma peritoneal metastases that are attenuated by YAP1 inhibition. Gut.

[9] He C, Lv X, Huang C (2019). A Human Papillomavirus-Independent Cervical Cancer Animal Model Reveals Unconventional Mechanisms of Cervical Carcinogenesis. Cell Rep.

[10] Zhou Q, Bauden M, Andersson R (2020). YAP1 Is an Independent Prognostic Marker in Pancreatic Cancer and Associated With Extracellular Matrix Remodeling. J Transl Med.

[11] Jiang YY, Lin DC, Mayakonda A (2017). Targeting super-enhancer-associated oncogenes in oesophageal squamous cell carcinoma. Gut.

[12] Lee KW, Lee SS, Kim SB (2015). Significant association of oncogene YAP1 with poor prognosis and cetuximab resistance in colorectal cancer patients. Clin Cancer Res.

[13] Masahiro S, Kendall H, Mohammad OH (2018). A time for YAP1: Tumorigenesis, immunosuppression and targeted therapy. Int J Cancer.

[14] Ho WJ, Jaffee EM, Zheng L The tumour microenvironment in pancreatic cancer — clinical challenges and opportunities. Nat Rev Clin Oncol.

[15] Karanikas M, Esempidis A, Chasan ZT (2016). Pancreatic Cancer from Molecular Pathways to Treatment Opinion. J Cancer.

[16] Morrison AH, Byrne KT, Vonderheide RH (2018). Immunotherapy and Prevention of Pancreatic Cancer. Trends Cancer.

[17] Banerjee K, Kumar S, Ross KA (2018). Emerging trends in the immunotherapy of pancreatic cancer. Cancer Lett.

[18] Tang Z, Li C, Kang B (2017). GEPIA: a web server for cancer and normal gene expression profiling and interactive analyses. Nucleic Acids Res.

[19] Anaya J (2016). OncoLnc: linking TCGA survival data to mRNAs, miRNAs, and lncRNAs. PeerJ Computer Science.

[20] Chirayu PG, Harikrishna N (2014). PROGgeneV2: enhancements on the existing database. BMC Cancer.

[21] Karen C, Susanne G (2011). A roadmap to generate renewable protein binders to the human proteome. Nat Methods.

[22] Suhas VV, Peter S, Wang J (2018). LinkedOmics: analyzing multi-omics data within and across 32 cancer types. Nucleic Acids Res.

[23] David WF, Sylva LD, Ovi C (2010). The GeneMANIA prediction server: biological network integration for gene prioritization and predicting gene function. Nucleic Acids Res.

[24] Newman AM, Liu CL, Green MR (2015). Robust enumeration of cell subsets from tissue expression profiles. Nat Methods.

[25] Eleonora L, Marco P, Pina Z (2019). New therapeutic targets in pancreatic cancer. Cancer Treat Rev.

[26] Lee CK, Jeong SH, Jang C (2019). Tumor metastasis to lymph nodes requires YAP-dependent metabolic adaptation. Science.

[27] Ameer LE, Arthur MM (2018). Convergence of VEGF and YAP/TAZ signaling: Implications for angiogenesis and cancer biology. Sci Signal.

[28] Qiao Y, Chen JX, Lim YB (2017). YAP Regulates Actin Dynamics through ARHGAP29 and Promotes Metastasis. Cell Rep.

[29] Liu P, Lu ZW, Liu LL (2019). NOD-like receptor signaling in inflammation-associated cancers: From functions to targeted therapies. Phytomedicine.

[30] Sachin HP, Fernando DC, Dean Y (2017). Hippo Signaling in the Liver Regulates Organ Size, Cell Fate, and Carcinogenesis. Gastroenterology.

[31] Bodo EL (2013). Cytokine patterns in patients with cancer: a systematic review. Lancet Oncol.

[32] Wang P, Zhang X, Sun N (2020). Comprehensive Analysis of the Tumor Microenvironment in Cutaneous Melanoma associated with Immune Infiltration. J Cancer.

[33] Zhang R, Liu Q, Peng J (2020). CXCL5 overexpression predicts a poor prognosis in pancreatic ductal adenocarcinoma and is correlated with immune cell infiltration. J Cancer.

[34] Chen EB, Zhou ZJ, Xiao K (2019). The miR-561-5p/CX 3 CL1 Signaling Axis Regulates Pulmonary Metastasis in Hepatocellular Carcinoma Involving CX 3 CR1 + Natural Killer Cells Infiltration. Theranostics.

[35] Pan JH, Zhou H, Laura C (2019). LAYN Is a Prognostic Biomarker and Correlated With Immune Infiltrates in Gastric and Colon Cancers. Front Immunol.

[36] Postow MA, Callahan MK, Wolchok JD (2015). Immune Checkpoint Blockade in Cancer Therapy. J Clin Oncol.

[37] Wang GC, Lu X, Prasenjit D (2016). Targeting YAP-Dependent MDSC Infiltration Impairs Tumor Progression. Cancer Discov.

[38] Dondi E, Rogge L, Lutfalla G (2003). Down-modulation of responses to type I IFN upon T cell activation. J Immunol.

