# Supplementary material for: Comprehensive analysis of gene regulation network and immune signatures of prognostic biomarker YAP1 in pancreatic cancer
Source: J Cancer. 2020 Oct 8;11(23):6960–9. doi: 10.7150/jca.49117 (PMC7592007; doi:10.7150/jca.49117)
Supplement: Supplementary file 1 — Supplementary figures and tables. [file jcav11p6960s1.pdf]

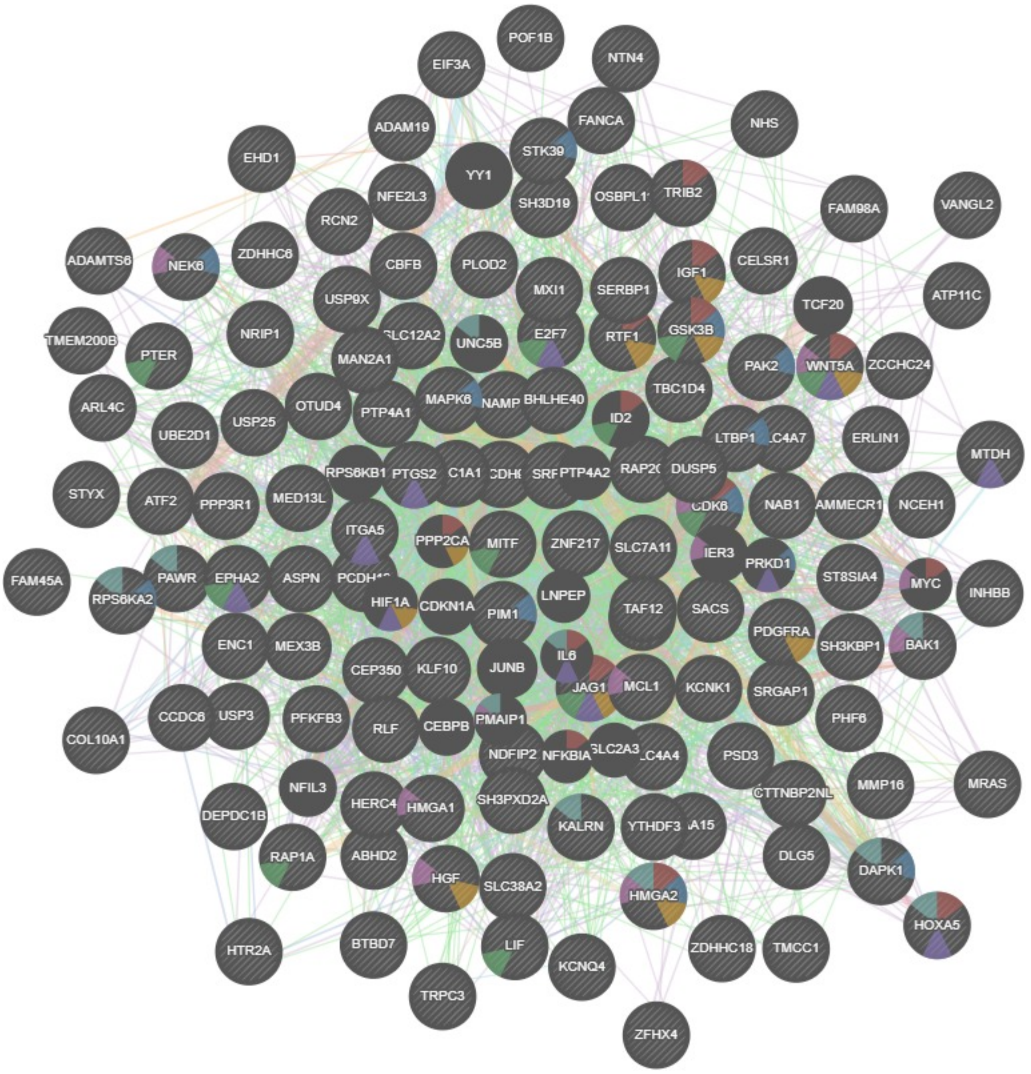

Networks

- Co-expression
- Physical Interactions
- Co-localization
- Genetic Interactions
- Pathway
- Predicted
- Shared protein domains

Functions

- negative regulation of cell differentiation
- protein serine/threonine kinase activity
- stem cell differentiation
- angiogenesis
- epithelial cell differentiation
- regulation of cellular response to stress
- positive regulation of programmed cell death

Figure.S1 PPI network of MiR-target networks (GeneMANIA). The biological functions of the gene sets of MiR-target networks were assessed using the applied bioinformatics methods via the PPI network.

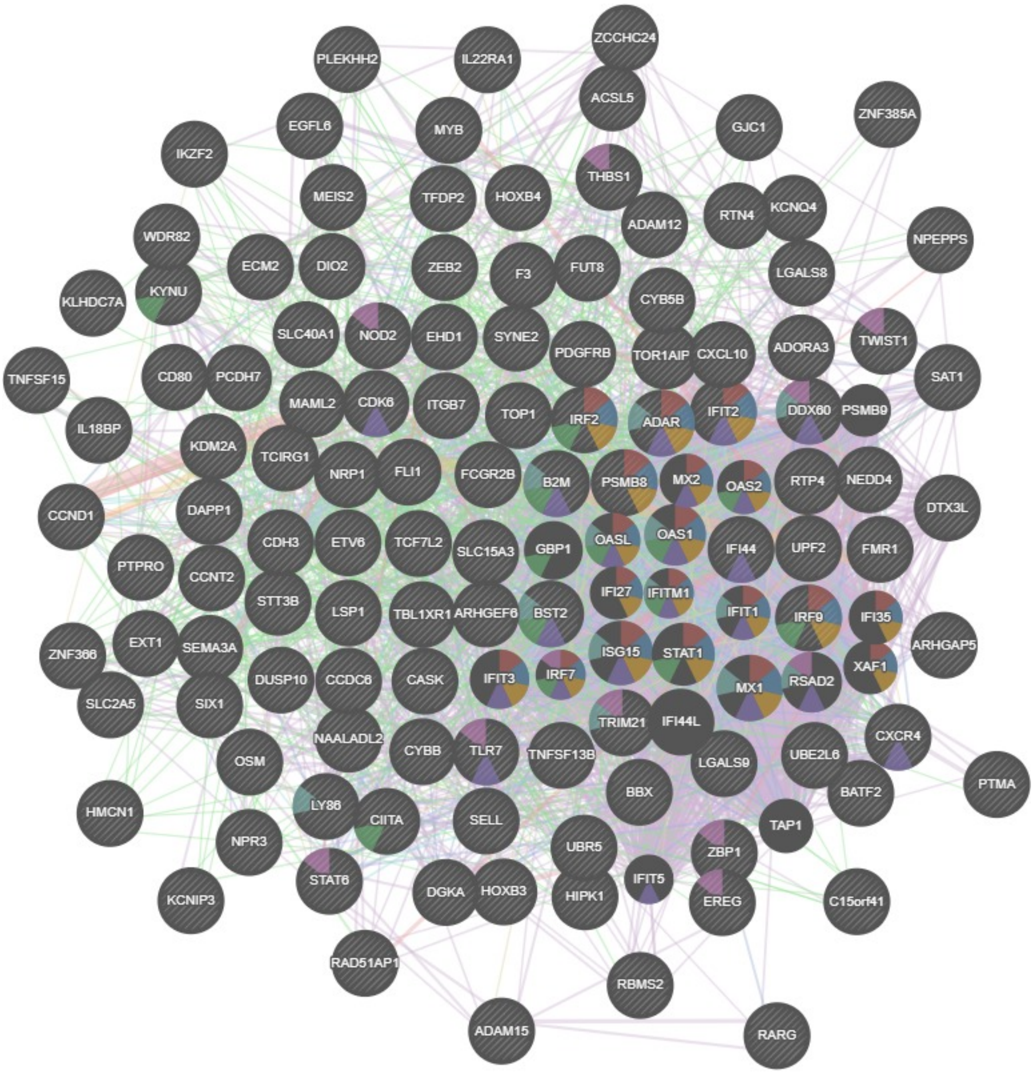

Networks

- Co-expression
- Physical Interactions
- Co-localization
- Genetic Interactions
- Predicted
- Pathway
- Shared protein domains

Functions

- response to type I interferon
- cellular response to type I interferon
- type I interferon signaling pathway
- response to virus
- response to interferon-gamma
- regulation of multi-organism process
- positive regulation of cytokine production

Figure.S2 PPI network of TF-target networks (GeneMANIA). The biological functions of the gene sets of TF-target networks were assessed using the applied bioinformatics methods via the PPI network.
